# Adult attention-deficit/hyperactivity symptoms and parental cognitions: a meta-analysis

**DOI:** 10.3389/fpsyt.2023.1321078

**Published:** 2024-01-09

**Authors:** Mónika Miklósi, Barbara Kovács, Júlia Janovicz, Franciska Lelki, Réka Kassai

**Affiliations:** ^1^Psychological Institute, Eötvös Loránd University, Budapest, Hungary; ^2^Department of Clinical Psychology, Semmelweis University, Budapest, Hungary; ^3^Centre of Mental Health, Heim Pál National Paediatric Institute, Budapest, Hungary; ^4^School of Doctoral Studies, Eötvös Loránd University, Budapest, Hungary

**Keywords:** attention/deficit hyperactivity disorder, ADHD, adult, dysfunctional cognition, parent, meta-analysis

## Abstract

**Introduction:**

Attention-deficit/hyperactivity disorder (ADHD) symptoms in adults interfere with parental functioning. Dysfunctional parental cognitions may play a role in this impairment. Despite the importance of parental cognitions on parents and children’s outcomes, up to now, no systematic review or meta-analysis of these findings is available. To fill this gap, this meta-analysis aimed to evaluate the relationship between adult ADHD symptoms and parental cognitions.

**Methods:**

We conducted searches in Web of Science, PubMed, and ProQuest from January 2000 to June 2023. Studies were included if they provided data on the relationship between parental ADHD symptoms and parental cognitions by means of a row correlational coefficient, or means and standard deviation were reported for each study group. A random-effects model was used. Publication bias was assessed by funnel plot and Rosenthal’s fail-safe *N*. Moderator analyses were conducted by means of subgroup analysis and meta-regression analyses.

**Results:**

Fifteen published papers were included (*N* = 2851), and 51 effect sizes were analysed. The weighted mean effect size was small but significant (Fisher’s *Z* = 0.186, *k* = 15, 95% CI [0.120 – 0.252], *z* = 5.539, *p* < 0.001), indicating that ADHD symptoms in adults are associated with more negative and less positive parental cognitions. The Fail-Safe *N* analysis suggested a robust effect. Tweedie’s trim and fill results suggested that five studies were missing; after five missing studies had been imputed, the mean overall effect size dropped to 0.116 (0.080 – 0.152). There was significant heterogeneity among effect sizes. The methodology of the study was found to be a significant moderator. Meta-regression analyses revealed that the lower age of the parent and the child were related to more negative parental cognitions.

**Discussion:**

Though the analysis might be inflated by publication bias, our results suggest a significant association between ADHD symptom level and dysfunctional parental cognitions. Biased negative perceptions of the parental role, the child and co-parenting may play a central mediator role between parental ADHD and parent and child outcomes. Given the familiar nature of ADHD, targeting dysfunctional parental cognitions in parent training programs is warranted.

**Systematic review registration:**

osf.io/pnur7.

## 1 Introduction

Attention-deficit/hyperactivity disorder (ADHD) (1) is one of the most prevalent chronic neuropsychiatric disorders evolving in childhood and continuing into adulthood in 4–77% of the cases (2). ADHD in adults has a worldwide prevalence of 2–3% (3, 4). About 70 to 75% of adults with ADHD are diagnosed with at least one comorbid mental disorder (5), e.g., mood and anxiety disorders (6), disruptive disorders (6), bipolar disorder (7), substance use and substance use disorders (8–10), behavioral addictions (11), insomnia (12), and personality disorders (13). In addition to the core symptoms of inattention and hyperactivity/impulsivity, emotional dysregulation (14), and executive function deficit (15, 16) are the characteristics of adult ADHD that lead to impaired functioning in multiple areas of life (17, 18) including the interpersonal domain (19–21).

Parenting is one of the important interpersonal functions ADHD symptoms in adults may interfere with (22). In their meta-analytic review, Park and Johnston (23) found that higher levels of ADHD symptoms in the parent are associated with less positive and more harsh and lax parenting behaviors. Effect sizes were small but robust across ADHD symptom clusters, parents’ gender, and children’s age. Furthermore, parental ADHD symptoms have been reported to be the strongest predictor of parenting stress, even after controlling for the child’s ADHD symptoms and oppositionality (24). Intervention research revealed that high levels of maternal ADHD symptoms undermine the effectiveness of behavioral parent training in parents of children with ADHD (25, 26). It has been suggested that cool and hot executive dysfunctions and self-regulation deficits may account for these impairments (27).

Self-regulation and underlying executive functions are thought to be fundamental to successful adaptation to the cognitively and emotionally demanding challenges of parenting (28). Information processing during parent–child interactions, regulation of negative emotions and inhibition of automatic reactions in stressful child-rearing situations, and flexible adaptation of emotional and behavioral responses to changing developmental demands require intact working memory capacity, inhibitory control, frustration tolerance, the ability to delay gratification, cognitive flexibility, self-monitoring, planning, problem-solving and organization skills (29–31). Less effective executive functioning was shown to be related to higher levels of harsh and lower levels of warm parenting (32) and risk of physical abuse through emotional dysregulation (33). Beyond the direct association between executive function deficit and negative parenting, there is some evidence of the moderating effect of inhibitory control on the relationship between parental hyperactive/impulsive symptoms and overreactive parenting, as well as inattention and lax parenting (34).

Deficits in self-regulation and executive functions not only affect behavior but also interact with environmental challenges in forming the individual’s views of the self and the world from the person’s formative years (35, 36). Consequently, more negative self-concepts and lower levels of general self-efficacy (37), and self-esteem (38), especially when untreated (39), have been reported in adults diagnosed with ADHD. According to narrative reviews, the self-concept of adults with ADHD could be characterized by maladaptive beliefs about the self, i.e., failure, impaired self-control, being different from others and a sense of inadequacy (40, 41). Furthermore, some evidence refers to higher levels of more situational negative automatic thoughts in adults with ADHD compared to healthy controls (41). Besides studies on negative thinking styles, there is a growing recognition that dysfunctional cognitions in adult ADHD may also be irrationally positive or optimistic (42).

It is plausible to assume that stressful child-rearing situations may trigger these dysfunctional cognitions in parents with ADHD, resulting in a biased negative perception of the parental role and the child. Repeated failure in parenting situations resulting from core deficits in ADHD and frequent negative feedback about the person’s parenting skills may also lead to increased parental stress and low parental self-efficacy which in turn may negatively affect the parent–child relationship and parenting behavior. In that way, dysfunctional parental cognitions may play a central mediator role between parental ADHD and parent and child outcomes (27). On the other hand, a positive bias by means of an overestimation of positive parenting behaviors in adults with ADHD (43) may lead to an irrationally increased parental self-efficacy.

Despite the importance of parental cognitions on parents’ and children’s functioning and the growing evidence of biased parental cognitions associated with adult ADHD, up to now, there is no systematic review or meta-analysis of these findings is available.

To fill this gap, this meta-analysis aimed to evaluate the relationship between adult ADHD symptoms and parental cognitions. We aimed to address this question in both dimensional and categorical approaches. More specifically, our research questions were: Are higher levels of ADHD symptoms in adults related to more negative and less positive parental cognitions? Do adults with ADHD report more negative and less positive parental cognitions than healthy or non-clinical controls? Based on the literature reviewed above, we hypothesized that higher levels of parental ADHD symptoms would be associated with more dysfunctional parental cognitions.

Further research questions were related to possible moderators: Does the relationship between adult ADHD symptoms and parental cognitions vary across the child’s age groups, parent’s gender, ADHD symptom clusters, and different types of cognitions: across cognitions about the self as a parent (i.e., parental self-efficacy beliefs, the perception of the parental role as rewarding or burdensome), the child (i.e., attitudes toward the child, attributions for the child’s behavior), and co-parenting; by valence of the cognition (negative/positive); and, by stability of the cognition (stable/situational)?

## 2 Materials and methods

Methods have been developed following the recommendations of the Preferred Reporting Items for Systematic Reviews and Meta-Analyzes (PRISMA) 2020 Statement (44). The protocol of the study has been preregistered at OSF.^1^

### 2.1 Inclusion and exclusion criteria

#### 2.1.1 Type of publication

Original studies published in peer-reviewed journals between January 2000 and May 2023, or dissertations/theses uploaded in repositories and available in full were considered. Only empirical studies were included, and case studies, case series, as well as studies applying qualitative methodologies were excluded. We also contacted some of the authors of existing papers for possible non-published studies.

#### 2.1.2 Population

Studies involving both clinical and non-clinical parent samples were included. We did not have any exclusion criteria regarding comorbidities, demographic or SES characteristics of the sample, or the geographic location of the study. Studies were included regardless of the past and current treatment of the participants.

#### 2.1.3 Outcome

The primary outcome was the relationship between parental ADHD symptom level and parental cognitions by means of a standardized correlational coefficient. The definition of ADHD was based on the relevant versions of the Diagnostic and Statistical Manual of Mental Disorders, fourth edition, text revision (DSM-IV-TR (45)), and fifth edition [DSM-5 (1)]. Predominantly inattentive, predominantly hyperactive/impulsive, and combined presentations were all included in the definition. Parental cognitions were defined as cognitions about parenting, the parental role, the self as a parent, the child, and co-parenting. The distinction between parental cognitions and behavior is not always clear; we included studies focusing on expectations, perceptions, attitudes, attributions, beliefs, and values, but excluded constructs that are traditionally referred to in the literature as parenting practices, parenting styles, or parenting behaviors (e.g., warmth, nurturance, overprotectiveness) even if they include a cognitive component. A distinction between parental cognitions and parenting stress defined as “aversive psychological and physiological reactions arising from attempts to adapt to the demands of parenthood” (46, page 6) was also made and studies assessing parenting stress were excluded.

Regarding the measurement of study variables, we had two criteria. First, regarding the measurement of ADHD, we included studies that assessed the actual severity of ADHD symptoms with a reliable and valid instrument (a structured clinical interview or a questionnaire) or established ADHD diagnosis in the clinical group with a reliable and valid structured or at least semi-structured clinical interview and assessed mental disorders in the comparison group by using the same procedure. Studies using a patient group with a self-reported ADHD diagnosis only, or a childhood diagnosis of ADHD without measuring current symptom severity, were excluded. Second, studies must have included a valid and reliable measure for the assessment of any type of parental cognitions defined above. Studies using self-report, partner-report, or behavioral observation were included.

In the end, for their inclusion, studies must have met one of the following criteria (1): the relationship between parental ADHD symptoms and parental cognitions was reported by means of a row correlational coefficient or (2) means and standard deviation were reported for both the ADHD diagnosed parent-group and for at least one comparison group (non-clinical or healthy controls, or a patient group with other mental disorders but not ADHD).

### 2.2 Search strategy

#### 2.2.1 Data-bases

Electronic searches were performed (BK, JJ) in the following databases: Web of Science, PubMed, and ProQuest including Dissertations and Theses.

#### 2.2.2 Keywords

Keywords for ADHD and parental cognitions were combined. The final search term is shown in Supplementary Table S1.

#### 2.2.3 Further specifications

Only English-language papers were included. The date of publication or submission year of the dissertation/thesis must have been between January 2000 and May 2023.

#### 2.2.4 Additional search

We conducted searches in the reference lists of previous review papers and in reference lists and citations of the papers found by the machine search.

### 2.3 Identification and selection of studies

Studies identified by electronic and manual searches – after removing duplicates – were evaluated by two independent researchers (KB, LF), according to their titles and abstracts. The final list was agreed upon, and discrepancies were resolved by consensus between the two researchers. The full-text version of the papers of the final list was downloaded and assessed for eligibility by two independent researchers (FL, JJ). Discrepancies were resolved by consensus between the two researchers. We linked together multiple reports from the same study, and for the same analysis, the highest quality report was considered (e.g., a published paper instead of a dissertation). From longitudinal studies, only baseline data was included.

### 2.4 Data extraction

The following data were extracted and inserted in an Excel sheet by two independent researchers (FL, JJ): publication details (citation, year, country); design (correlational, comparison of multiple groups); study participants, sample size, mean age of parents, % of mothers in the parent sample, mean age of children, children’s age range, % of boys in the sample, sample characteristics (populational/clinical, type of comparison group, comorbid characteristics of clinical groups); method to establish parents’ ADHD diagnosis and/or assessment of adult ADHD symptoms, cluster of ADHD symptoms measured (inattention, hyperactivity/impulsivity, combined); characteristics of parental cognitions assessed: valence (negative/positive), stability (stable/situational), reference (self/child/co-parenting), domain (self-efficacy/role/attitude/attribution), method (self-report/partner-report/observational), measure. Data from measures assessing positive cognitions were recoded, in that way higher scores represented lower levels of positive cognitions.

### 2.5 Assessment of study quality and bias

Study quality and bias assessment were conducted by two independent researchers (MM, BK) by using the modified version Effective Public Health Practice Project (EPHPP) Quality Assessment Tool for Quantitative Studies (47). Discrepancies were resolved by consensus between the two researchers.

### 2.6 Statistical analyzes

The Comprehensive Meta-analysis (48) software was used for the analysis. We used random effect models which include sampling and study-level errors. All effect sizes were transformed to Pearson’s correlational coefficients, which then was standardized using Fisher’s transformation. The overall effect size was calculated and reported as Fisher’s *Z* value. The heterogeneity between studies was tested with Cochran’s *Q* test and with *I^2^* values (0–40%: not important; 30–60%: moderate, 50–90% substantial, 75–100%: considerable heterogeneity). Publication bias was assessed visually by funnel plot and Rosenthal’s fail-safe *N* (49). If publication bias was detected, we adjusted for this using Duval Tweedie’s method (48).

Moderator analyzes were conducted by means of subgroup analysis in case of categorical moderators: the children’s age groups, the parent’s gender, ADHD symptom clusters, stability of the cognition (stable/situational), by the valence of the cognition (positive/negative), across cognitions about the self as a parent (i.e., parental self-efficacy beliefs, the perception of the parental role as rewarding or burdensome), the child (i.e., attitudes toward the child, attributions for the child’s behavior), and co-parenting; and the method of assessing parental cognitions (observation, self-report, partner-report). We conducted meta-regression analyzes of the moderating effect of publication year, study quality, the mean age of children and parents, the ratio of boys, and the ratio of mothers in the sample. The stability of the results and the influence of studies were tested using leave-one-study-out sensitivity analysis. Effect sizes were tested for potential outliers and standardized residuals over +/− 3.29 were excluded.

## 3 Results

### 3.1 Study selection

**Figure 1:**
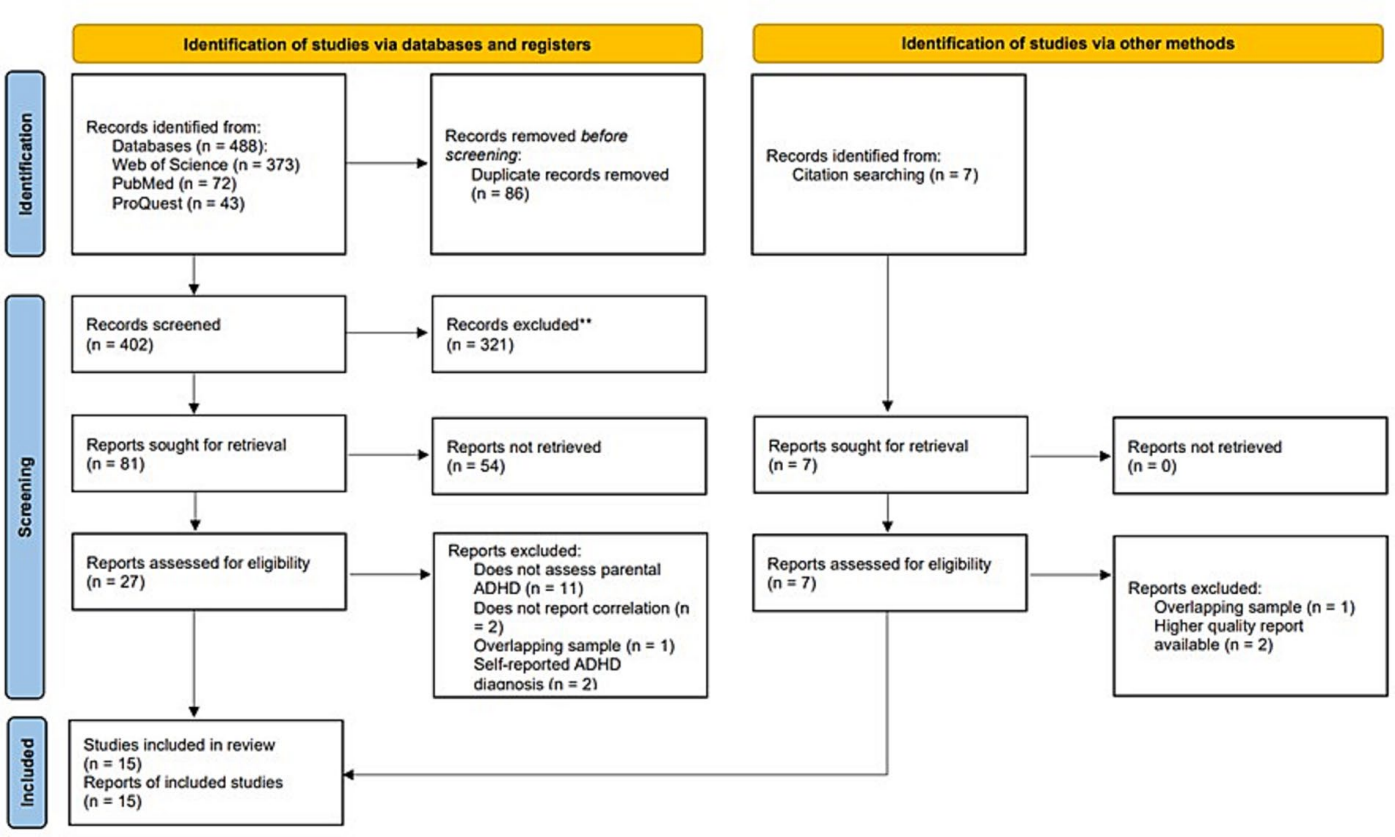
PRISMA flow-diagram (44).

The flowchart of the eligible studies is represented in Figure 1. In three databases we identified 488 records. After removing duplicates, 402 records were screened by title, from which 81 papers were sought for retrieval and 27 full texts were assessed for eligibility criteria. Eleven studies did not assess parental ADHD (50–60), two studies did not report the correlational coefficients between parental cognition and ADHD symptoms (61, 62), and two studies used self-reported ADHD diagnosis (63, 64), therefore these studies were excluded. After contacting the authors we excluded an additional study (65) because its sample was highly overlapping with another study of the same research group (66). We conducted searches in citations and references of existing papers and identified seven additional records. They were all assessed for eligibility. According to the authors contacted, one study (67) used the same sample as a previously included paper (68), and there were two dissertations (69, 70) among the records for which the published versions were also identified. These three records were excluded. Taken together, 11 papers from the database search and 4 additional papers from the citation and reference search were included (Figure 1).

Fifty-one effect sizes of 15 published papers were included in the analyzes (*N* = 2,851). For study characteristics, see Table 1.

**Table 1:** Study characteristics.

Study		Parents’ characteristics		Children’s characteristics				Measure			
First author, year (Country)	Sample size	Mean age (years)	Mothers (%)	Boys (%)	Mean age (years)	Age range (years)	ADHD diagnosis (%)	ADHD measure	ADHD symptom cluster	Cognition measure	Method
Banks et al. (2008) (Canada) (77)	80	32.3	100	–	–	3–6	–	ABCA/CSS, CAARS	C	PLOC, PSOC	SR
Fabrikant-Abzug et al. (2023) (United States) (68)	199	–	89.5	58	8.6	7–11	100	CAARS, ASEBA/ASR	C	PCEQ	SR
Johnston et al. (2018) (Canada) (66)	156 dyads	43.1	50	100	9.6	5–13	70.5	BAARS-IV	IA, H/I	CRI, IRI	SR
Lindström et al. (2022) (Sweden) (82)	549	43.3	61	70.1	10.14	3–17	100	ASRS	C	WAQ	SR
Lowry et al. (2018) (United States) (71)	79 dyads	41.1	50	71.1	8.5	6–12	100	ASRS	C	PSOC CGSQ	SR, PR
Moroney et al. (2017) (United States) (81)	205	–	87	68.0	10.2	7–12	53	ASRS	C	FMSS	O
Ninowski et al. (2007) (Canada) (78)	86	31.1	100	NA	NA	NA	NA	CAARS, ABCA/CSS	C	PMES	SR
Park et al. (2019) (Canada) (75)	79	–	100	100	–	6–12	–	BAARS-IV	C	ARS	SR
Psychogiou et al. (2007) (United Kingdom) (79)	100	–	100	100	7.9	School-aged	–	AARS	C	FMSS	O
Psychogiou et al. (2008) (United Kingdom) (72)	268	–	100	57	7.7	School-aged	–	AARS	C	IRI	SR
Richards et al. (2014) (NL) (73)	385	–	100	83.4	11.5	5–18	100	ADHD-RS-IV	C	CFI	O
Sonuga-Barke et al. (2002) (United Kingdom) (83)	83	–	100	63.4	3	NA	100	AARS	C	PSOC	SR
Watkins et al. (2009) (Canada) (80)	99	33.0	100	35.4	0.5	NA	–	CAARS	C	PACOTIS PSOC	SR
Williamson et al. (2016) (Canada) (74)	64 dyads	–	50	100	9.6	8–12	41.0	ABCA/CSS	C	PAM	SR
Williamson et al. (2019) (Canada) (76)	120	33.9	100	57.0	7.8	6–12	–	BAARS-IV	C	P-SEMI	Coll, SR

ABCA, ADHD Behavior Checklist for Adults; AARS, Adult AD/HD Rating Scale; BAARS-IV, Barkley Adult ADHD Rating Scale–IV; ASRS, Adult ADHD Self-Report Scale; CAARS, Conners’ Adult ADHD Rating Scales; ASR, Adult Self Report, Attention Problems; ADHD-RS-IV, ADHD Rating Scale-IV; IA, Inattention; H/I, Hyperactivity/Impulsivity; C, Combined; PSOC, Parental Sense of Competence Scale; PMES, Prenatal Maternal Expectations Scale; ARS, Attribution Rating Scale; CGSQ, Caregiver Strain Questionnaire; CRI, Child Rearing Inventory; IRI, Interpersonal Reactivity Index; PCEQ, Parental Cognitive Error Questionnaire; WAQ, Written Analog Questionnaire; FMSS, Five-Minute Speech Sample; PAM, Parenting Alliance Measure; PLOC, Parental Locus of Control Scale; PACOTIS, Parental Cognitions and Conduct Toward the Infant Scale; P-SEMI, Parenting Sense of Efficacy Instrument; CFI, coding system of the Camberwell Family Interview; SR, self-report. PR: partner-report; Coll, Collateral Informants; O, observation; CoP, Co-parenting; NA, non-applicable.

### 3.2 Study characteristics

#### 3.2.1 Quality of studies

Most of the studies involved can be rated as studies with strong quality assessment, but there are some points where we found some weaker rates (Supplementary Table S2). In connection with the aspects of selection bias, there are 6 studies in which we cannot tell what percentage of selected individuals have agreed to participate (66, 68, 71–74). In two studies there are questions about the selection of the appropriate target population (75, 76). Questions regarding the blinding procedure show that we have one study in which they do not provide any information about the awareness of the participants (77). According to other aspects strong ratings could be given.

#### 3.2.2 Samples

Seven studies involved community samples of parents (72, 75–80). Three studies’ participants were parents of children with and without ADHD diagnosis (66, 74, 81) and five studies involved only parents of children with ADHD (68, 71, 73, 82, 83). Two studies reporting group comparisons (77, 83) grouped community samples of parents according to varying levels of ADHD symptoms. Eight studies involved only mothers, and mothers were overrepresented in almost all samples except for three studies involving mother–father dyads (66, 71, 74). Four studies involved only parents of boys, and boys were overrepresented in six further samples. According to the age of children, a single study involved first-time expectant women (77, 78), one study involved parents of 6-month-old infants (79, 80), two studies involved parents of preschool-aged children, eight studies involved parents of school-aged children, and three studies used mixed samples.

#### 3.2.3 Measures for parental ADHD

All studies used reliable and valid rating scales for assessing current parental ADHD symptom levels. Three studies used the Current Symptom Scale of the ADHD Behavior Checklist for Adults [ABCA (84)], three measured parental ADHD symptoms with the Adult AD/HD Rating Scale [AARS (85)], three studies reported ADHD symptoms according to the Barkley Adult ADHD Rating Scale–IV [BAARS-IV (86)], and four studies used the Conners’ Adult ADHD Rating Scale [CAARS (87)]. The Adult ADHD Self-Report Scale [ASRS (88)] was used in three studies. Most of the studies used the total scores of the scales, only a single study reported results for inattention and hyperactivity/impulsivity symptom scores separately. A single study used composite scores of the Adult Self Report, Attention Problems subscale (ASR) of the Achenbach System of Empirically Based Assessment [ASEBA (89)] and the Conners’ Adult ADHD Rating Scale. One study used collateral informants, and another study used both self- and partner-reports, all other studies assessed ADHD symptoms using self-report.

#### 3.2.4 Measures for parental cognitions

A variety of parental cognitions have been explored in the studies, including parental beliefs about the self as a parent (parental self-efficacy beliefs and expectations of and satisfaction with the parental role), attitudes toward the child (tolerance of misbehavior, parental critique, and empathy toward the child), and attributions of child (mis)behavior (controllability, intentionality, responsibility, perceived parental impact, locus of control). Most studies used self-report measures of parental cognition. One study gathered additional information from the partner, and three studies conducted behavioral observation.

Parental self-efficacy, i.e., the degree to which parents perceive themselves as capable of performing tasks associated with the parental role (90), was the most frequently assessed construct. It was measured by the Parental Sense of Competence Scale [PSOC (91, 92)] in four studies, but the Parental Cognitions and Conduct Toward the Infant Scale [PACOTIS (93)], the Parenting Sense of Efficacy Instrument (P-SEMI (94)), and the Parental Locus of Control Scale [PLOC (95)] were also used to evaluate parental self-efficacy. The PSOC was also used to assess the degree of satisfaction derived from the parenting role. The perception of strain related to one’s role as a caregiver was measured by the Caregiver Strain Questionnaire (CGSQ (96)), and the prenatal expectations regarding the infant and the future maternal role were assessed by the Prenatal Maternal Expectations Scale (PMES (97)).

Causal attributions about the child’s undesirable behavior were assessed by the modified Written Analog Questionnaire [WAQ (92, 98)] and the Attribution Rating Scale [ARS (75)]. The PLOC (95) was also used to assess whether parents view their child’s behavior as a direct consequence or outside the reach of their parenting efforts. A single study measured cognitive distortions related to attributions of negative child behavior and parenting by the Parental Cognitive Error Questionnaire [PCEQ (99)].

Attitudes toward the child, i.e., tolerance of misbehavior and parental empathy toward the child, were assessed by the Child Rearing Inventory (CRI (100)), and the Interpersonal Reactivity Index (IRI (101, 102)).

Two studies using behavioral observation assessed the parent’s perception of the child and their relationship by the Five-Minute Speech Sample [FMSS (103)]. In one study, relational schemas about the child were assessed during the structured clinical interviews using the coding system of the Camberwell Family Interview (104).

Cognitions about the alliance in raising a child with another parent were assessed in a single study using the Parenting Alliance Measure [PAM (105)].

### 3.3 Main analysis

#### 3.3.1 Mean effect size

**Figure 2:**
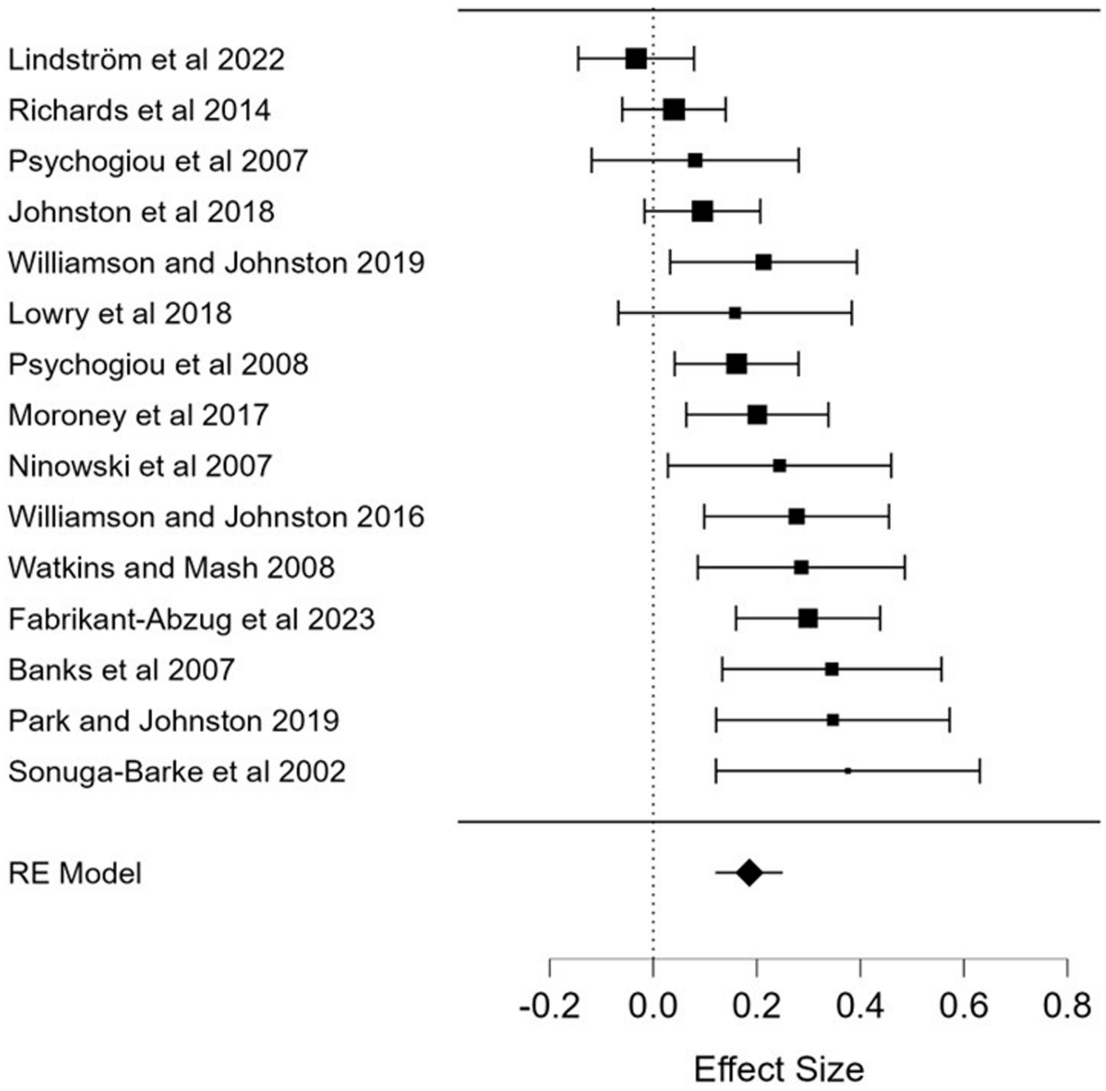
Forest plot of the associations of adult ADHD symptoms and parental cognitions. RE, random effect. Effect size: Fisher’s Z.

Across 15 studies, standardized residuals fell between −2.03 and 1.19 suggesting no outliers. The weighted mean effect size was small but significant [Fisher’s *Z* = 0.186, 95% CI (0.120–0.252), *z* = 5.539, *p* < 0.001], indicating that ADHD symptoms in adults are associated with more negative and less positive parental cognitions. Effect sizes ranged from −0.033 to 0.376, with all but one effect size in the expected direction and 10 of 15 effect sizes reaching statistical significance (Figure 2). Homogeneity analyzes indicated that there was a significant heterogeneity among effect sizes [*Q*(14) = 35.373, *p* = 0.001, *I^2^* = 60.422].

Sensitivity analyzes were performed to test the robustness of the effect by omitting one study at a time from the random-effect model. Mean effect sizes fell between 0.176 and 0.202 indicating a robust effect.

#### 3.3.2 Publication bias

**Figure 3:**
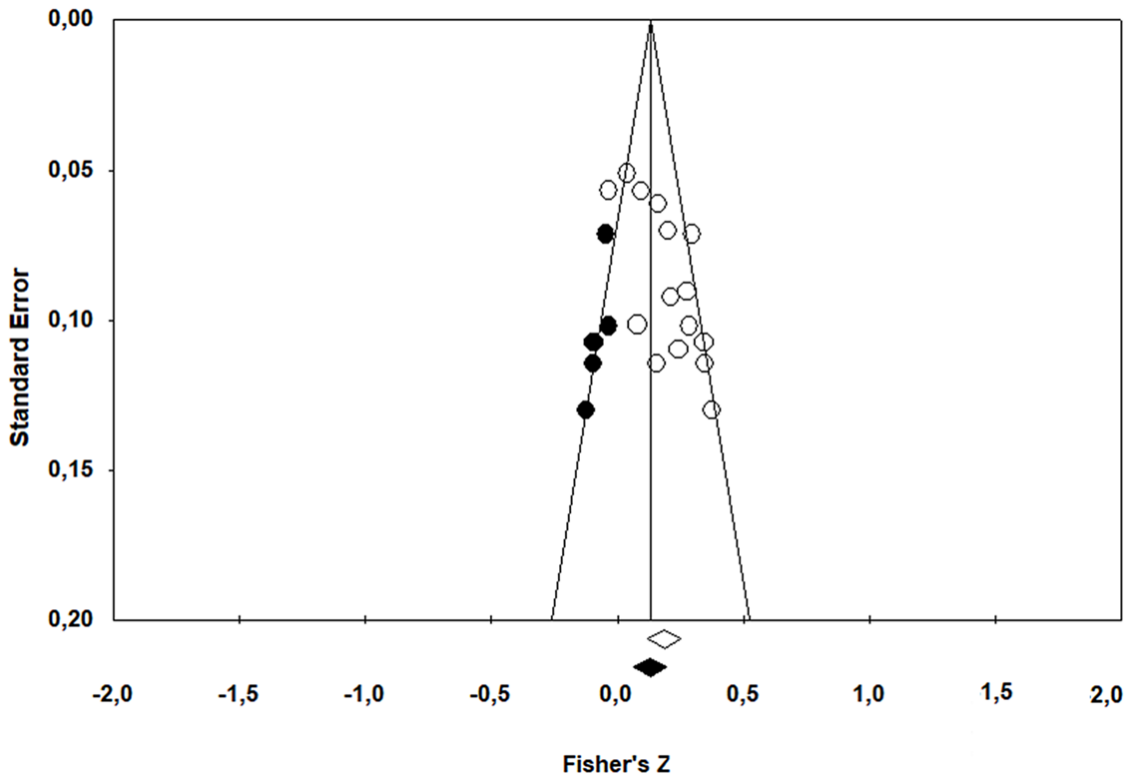
Funnel plot after trim and fill.

The Fail-Safe *N* analysis revealed that approximately 273 additional studies would be needed to bring the overall effect size for the association between adult ADHD symptoms and parental cognitions to a non-significant level, which is larger than the tolerance level of 5 * *k* + 10 = 85, suggesting a robust effect. A funnel plot of observed and imputed studies is shown in Figure 3. Tweedie’s trim and fill results suggested that five studies were missing. Using trim and fill, after five missing studies had been imputed, the mean overall effect size dropped to 0.116 [0.080–0.152]. The rank correlation coefficient, Kendall’s tau was 0.43, *p* = 0.023, significant and Egger’s regression method produced an intercept of 3.779, which was also significant (*p* = 0.002), supporting a conclusion that publication bias was operating. Taken together, these analyzes suggest that, while the results might be inflated by publication bias, the adjusted mean effect size continues to show that there is a significant association between ADHD symptom level and dysfunctional parental cognitions.

### 3.4 Subgroup analyzes

#### 3.4.1 Children’s age groups

The association between adult ADHD symptoms and parental cognitions were significant across all age groups of children. The effect size was small in a single study involving pregnant women [Fisher’s *Z* = 0.244, *k* = 1, 95% CI (0.029–0.459), *z* = 2.220, *p* = 0.026], similar to another single study in mothers of six-months-old infants [Fisher’s *Z* = 0.286, *k* = 1, 95% CI (0.086–0.486), *z* = 2.801, *p* = 0.005]. The weighted mean effect size of two studies that involved parents of preschool-aged children was 0.358, *k* = 2, 95% CI [0.195–0.520], *z* = 4.317, *p* < 0.001, representing a medium effect. Across eight studies in parents of school-aged children, the weighted mean effect size was small [Fisher’s Z = 0.214, *k* = 8, 95% CI (0.157–0.272), *z* = 7.275, *p* < 0.001], with a nonsignificant heterogeneity [*Q*(7) = 5.954, *p* = 0.545, *I^2^* = 0.000]. Taken together, these results indicated that higher levels of parental ADHD symptoms were associated with dysfunctional parental cognitions across the child’s age.

#### 3.4.2 Parents’ gender

Only two studies reported results separately for mothers and fathers. The weighted mean effect sizes were not significant for mothers [Fisher’s *Z* = 0.075, *k* = 2, 95% CI (−0.225–0.375), *z* = 0.491, *p* = 0.624], with a significant heterogeneity [*Q*(1) = 4.123, *p* = 0.042, *I^2^* = 75.747], and was small for fathers [Fisher’s Z = 0.269, *k* = 2, 95% CI (0.135–0.403), z = 3.940, *p* < 0.001], with a nonsignificant heterogeneity [*Q*(1) = 0.138, *p* = 0.711, *I^2^* = 0.000].

#### 3.4.3 Attention-deficit/hyperactivity disorder symptom clusters

Only a single study reported results separately for attention-deficit and hyperactivity/impulsivity symptoms. The mean effect sizes were not significant for both separate symptom clusters [Fisher’s *Z* = 0.089, *k* = 2, 95% CI (−0.023–0.201), *z* = 1.561, *p* = 0.119 and Fisher’s *Z* = 0.101, *k* = 2, 95% CI (−0.011–0.213), *z* = 1.774, *p* = 0.076, respectively]. The weighted mean effect size for studies using composite scores of the two ADHD symptom clusters was small, but significant [Fisher’s *Z* = 0.197, *k* = 14, 95% CI (0.125–0.268), *z* = 5.367, *p* < 0.001]. The heterogeneity was significant [*Q*(13) = 34.169, *p* = 0.001, *I^2^* = 61.954].

#### 3.4.4 Stable versus situational cognitions

Nine studies assessed stable cognitions, the weighted mean effect size was small [Fisher’s *Z* = 0.233, *k* = 9, 95% CI (0.169–0.297), *z* = 7.146, *p* < 0.001], with a nonsignificant heterogeneity [*Q*(8) = 5.739, *p* = 0.676, *I^2^* = 0.000], and six studies focused on more situational cognitions, the weighted mean effect size was small [Fisher’s *Z* = 0.134, *k* = 6, 95% CI (0.030–0.238), *z* = 2.530, *p* = 0.011], but the heterogeneity was significant [*Q*(5) = 20.143, *p* = 0.001, *I^2^* = 75.177].

#### 3.4.5 Negative versus positive cognitions

Eight studies assessed negative parental cognitions. For these outcomes, the mean effect size was 0.145 [*k* = 8, 95% CI (0.048–0.241), *z* = 2.937, *p* = 0.003], and the heterogeneity was significant [*Q*(7) = 21.470, *p* = 0.003, *I^2^* = 67.396]. Ten studies assessed positive parental cognitions, the mean effect size was 0.269 [*k* = 10, 95% CI (0.179–0.359), *z* = 5.876, *p* < 0.001], the heterogeneity was also significant [*Q*(9) = 22.037, *p* = 0.009, *I^2^* = 59.160].

#### 3.4.6 Self-referent cognitions

Across six studies, the association between self-referent cognitions and parental ADHD symptoms was significant, indicating that higher levels of the symptoms are related to more negative cognitions about the self. The weighted mean effect size was small/medium [Fisher’s *Z* = 0.287, *k =* 6, 95% CI (0.201–0.373), *z* = 6.551, *p* < 0.001], the heterogeneity was not significant [*Q*(5) = 2.520, *p* = 0.773, *I^2^* = 0.000]. More specifically, the weighted mean effect size for the relationships between parental sense of competence and adult ADHD symptoms was 0.331 [*k* = 6, 95% CI (0.220–0.442), *z* = 5.848, *p* < 0.001] indicating a medium effect. The heterogeneity of the effect was not significant [*Q*(5) = 8.264, *p* = 0.142, *I^2^* = 39.500].

#### 3.4.7 Cognitions about the child

Across nine studies, the weighted mean effect size for the association for parental ADHD symptoms and cognitions about the child was 0.125 [95% CI (0.054–0.197), *k =* 9, *z* = 3.445, *p* = 0.001, *Q*(8) = 17.148, *p* = 0.029, *I^2^* = 53.346], representing a small effect. When analyzing different types of child-referent cognitions separately, results revealed, that, across six studies, the weighted mean effect size indicated that higher levels of ADHD symptoms in the parent are associated with more negative parental attitudes toward the child [Fisher’s *Z* = 0.120, 95% CI (0.061–0.179), *k* = 6, *z* = 3.980, *p* < 0.001], with a nonsignificant heterogeneity [*Q*(5) = 5.824, *p* = 0.324, *I^2^* = 14.149]. The weighted mean effect size was small, however. Across three studies, parental attributions about the child’s behavior were not significantly related to parental ADHD symptoms [Fisher’s *Z* = 0.158, *k* = 3, 95% CI (−0.084–0.399), *z* = 1.280, *p* = 0.200], with a significant heterogeneity among the effect sizes [*Q*(2) = 10.590, *p* = 0.005, *I^2^* = 81.114].

#### 3.4.8 Cognitions about co-parenting

Only a single study reported the relationships between adult ADHD symptoms and cognitions about co-parenting, effect size was small but significant [Fisher’s *Z* = 0.277, 95% CI (0.100–0.455), *z* = 3.061, *p* = 0.002], indicating that parents with higher levels of ADHD symptoms have a more negative perception of their collaboration in raising a child with another parent.

#### 3.4.9 The method of assessing parental cognitions

Across three observational studies, the weighted mean effect size for the association of adult ADHD symptoms and parental cognitions was nonsignificant [Fisher’s *Z* = 0.102, *k* = 3, 95% CI (−0.003–0.207), *z* = 1.908, *p* = 0.056], with a nonsignificant heterogeneity [*Q*(2) = 3.428, *p* = 0.180, *I^2^* = 41.660]. Twelve studies used self-report measures for assessing parental cognitions, the weighted mean effect size was 0.218 [*k* = 12, 95% CI (0.140–0.297), *z* = 5.429, *p* < 0.001], with a significant heterogeneity [*Q*(11) = 28.859, *p* = 0.002, *I^2^* = 61.883], representing a small but significant effect. Only a single study used a partner report, the effect was non-significant [Fisher’s *Z* = 0.065, 95% CI (−0.159–0.290), *z* = 0.570, *p* = 0.569].

### 3.5 Meta-regression analyzes

Meta-regression analyzes revealed that publication year (*b* = −0.006, *SE* = 0.005, *z* = −1.06, *p* = 0.290), quality rating (*b* = 0.004, *SE* = 0.022, *z* = 0.17, *p* = 0.867), the ratio of boys (*b* = −0.001, *SE* = 0.002, *z* = −0.78, *p* = 0.434), and ADHD diagnoses in children (*b* = −0.001, *SE* = 0.001, *z* = −1.54, *p* = 0.125), and the ratio of mothers in the sample (*b* = 0.004, *SE* = 0.002, *z* = 1.54, *p* = 0.124) did not have a significant effect, while the effects of parents’ mean age (*b* = −0.017, *SE* = 0.008, *z* = −2.18, *p* = 0.029) and the mean age of children (*b* = −0.021, *SE* = 0.009, *z* = −2.39, *p* = 0.017) were significant. The lower mean age of the parent and the child were related to more negative parental cognitions.

## 4 Discussion

### 4.1 Parental ADHD symptoms and dysfunctional cognitions

Parental beliefs and expectations about the parental role, the parents’ attitudes toward the child and their causal attributions about the child’s behavior play a potentially important role in shaping developmental trajectories (106). A growing body of research reported that ADHD symptoms in adults are associated with dysfunctional cognitions in general (37–39, 42, 107–110), and more specifically, in the parenting domain (66, 68, 71–75, 80–83). However, our meta-analysis was the first that aimed to assess the relationships between parental ADHD symptoms and parental cognitions.

We were able to include 15 studies of overall strong quality. As hypothesized, the analysis revealed a significant association between parental ADHD symptoms and dysfunctional parental cognitions; parents with higher levels of ADHD symptoms reported less positive and more negative parental cognitions. The weighted mean effect size was small, however. Though the analysis suggested that a publication bias may inflate the results, the effect was robust and remained significant after five missing studies had been imputed.

It is important to note that, though previous research found overly optimistic dysfunctional automatic thoughts about efficacy and performance in adult ADHD, leading to procrastination and avoidance (42), our meta-analysis did not provide any evidence for positively biased parental cognition. On the contrary, ADHD symptoms were related to less positive cognitions about the self as a parent.

The results suggest that stressful child-rearing situations may trigger dysfunctional cognitions in parents with ADHD, resulting in a biased negative perception of the parental role, the child and co-parenting. Repeated failure in parenting situations resulting from emotional dysregulation and executive function deficits related to ADHD (14–16) and frequent negative feedback about the person’s parenting skills may also lead to increased parental stress and negative cognitions, which in turn may negatively affect the parent–child relationship and parenting behavior (106). In that way, dysfunctional parental cognitions may play a central mediator role in the relationship between parental ADHD symptoms and parent and child outcomes.

ADHD in adults is often accompanied by other mental disorders (5). In this meta-analysis, we could not statistically control for comorbid symptoms, but the results of individual studies suggest that comorbid conditions do not fully explain the relationship between parental ADHD symptoms and dysfunctional parental cognitions. For example, Ninowski et al. (78) found that, after controlling for comorbid symptoms, ADHD symptoms still predicted less positive expectations about the infant and the future parental role in a sample of first-time expectant women. The results suggest that the relationship between parental ADHD and dysfunctional cognitions is not exclusively mediated by comorbid symptoms.

Not only do parental characteristics affect parental cognitions, but they also may be driven by the child’s characteristics. Previous meta-analyzes indicated that genetically influenced behaviors in the child affect and shape parental behavior (111), and, more specifically, externalizing symptoms in the child elicit changes in parents’ psychological stress and parenting practices (112). A recent study found that ADHD polygenic scores in the child significantly predicted lower levels of parental involvement and monitoring and higher levels of inconsistent discipline through the child’s ADHD symptoms after controlling for parental ADHD symptoms (113). It is plausible to assume that this evocative effect also exists in relation to parental cognitions. We could not control our analyzes for the child’s ADHD symptoms. However, Psychogiou and et al. (72) reported a significant negative association between parental empathy toward the child and the child’s ADHD symptoms, even when parental ADHD symptoms were included in the model, suggesting that child-driven effects might also operate on parental cognitions. Therefore, both the child’s and the parent’s characteristics should be incorporated into explanatory models of parental cognitions.

Because of the heterogeneity of the effects, we conducted several subgroup analyzes and meta-regressions to uncover factors affecting the relationship between parental ADHD symptoms and parental cognitions. The effect was small but significant across all age groups of children, for both stable and situational cognitions, for negative and positive cognitions, and for cognitions about the self, the child, and co-parenting. Across six studies assessing parental sense of competence, the weighted mean effect size reached the medium level. According to previous research, low general self-efficacy may be a central maladaptive belief in adults with ADHD (39). On the other hand, general self-efficacy was shown to be the strongest predictor of parental self-efficacy (114). In that way, lower levels of perceived parental competence may be related to more general beliefs about the person’s ability to meet responsibilities in different roles in life. Parental self-efficacy beliefs were shown to be related to several positive parent and child outcomes (115, 116) and served as mediators of treatment effects on parenting (117). Therefore, addressing dysfunctional cognitions about the parenting role and the person’s abilities to raise a child may be crucial in parent interventions when working with parents with ADHD symptoms.

Meta-regression analyzes indicated that the lower age of the parent and the child were related to more negative parental cognitions. Previous research revealed mixed evidence on age-related changes in parental cognitions. The older age of the parent was shown to be related to higher satisfaction with the parental role (118) but not to higher levels of parental self-efficacy (119). However, ADHD symptoms were shown to decline with age (120) in both the parent and the child, which may explain the decrease in the strengths of the association between parental ADHD symptoms and dysfunctional cognitions in our analysis.

The methodology of the studies was found to be an important moderator. Across 12 studies using self-report measures for assessing parental cognitions, the weighted mean effect size was small but significant. However, the association between parental ADHD symptoms and dysfunctional cognitions were non-significant across three observational studies and in a single study using partner report. This is in line with the results of a meta-analysis on the relationship between adult ADHD symptoms and parenting behavior (23), in that Park and Johnston found a larger effect in studies using self-report for both ADHD symptoms and parenting behavior than in studies using other methodologies. Though self-report is undoubtedly the most valid source of information about the individual’s beliefs, attitudes and attributions, self-report measures are susceptible to measurement error, especially social desirability biases. Self-report on ADHD symptoms may also be biased by executive function deficits and comorbid conditions such as depressive symptoms. On the other hand, behavioral observation may be prone to reactivity bias. Our results draw attention to the need for multi-method, multi-informant research in parental psychopathology, dysfunctional cognitions, and parental functioning.

### 4.2 Limitations and call for further research

Several factors might impact our results, limit the generalizability of the findings, and call for further research.

#### 4.2.1 Biased samples

The studies involved in the meta-analyzes used community samples or parents of children with ADHD, but none of them used adult ADHD samples. The limited range of ADHD symptoms displayed in the parents in these samples might contribute to the overall small effect sizes found in the analyzes. Further research is warranted on parents with a clinical diagnosis of ADHD.

Mothers were overrepresented in most of the samples. In previous studies, gender differences have been reported in symptom presentation, prevalence, comorbid profile, and social perception of ADHD symptoms (121, 122). Women may be more likely to show symptoms of inattention rather than hyperactivity/impulsivity, which may lead to delayed referral and diagnosis (122, 123). Furthermore, mothers and fathers may differ in their perception and parenting of a child with ADHD (124). These gender differences might impact the results of our analyzes; future research should focus on other caregivers as well.

Furthermore, boys were overrepresented in most samples. However, gender differences were reported in symptom presentation (125), etiology (126), referral (127), and, more importantly to our topic, in parental perception of ADHD (128); in that way, it is plausible to assume that child’s gender may impact parental cognitions.

#### 4.2.2 Studies from high-income countries

We included only English language publications, which is a clear limitation of the study. Perhaps related to this, two-thirds of the studies involved in the meta-analysis were conducted in North America, and one-third of them in North and Western Europe, in that way all of them came from high-income countries. Though the prevalence of ADHD is similar across countries with different levels of income, according to a recent narrative review (129), access to treatment, especially to psychological interventions, is overall limited in low and medium-income countries, which might have an impact on parents’ perception of the symptoms of and knowledge about ADHD and effective parenting. Cultural differences have also been reported in the structure of ADHD symptoms (130), and therefore, more research is needed in different cultural contexts.

#### 4.2.3 Constructs and measurement issues

Another limitation of the meta-analysis was that the concepts in the field of parental cognitions are sometimes overlapping and not well-defined (106); it was difficult to draw a conclusion across studies using varying constructs and measures of parental attitudes, attributions, and beliefs. On the other hand, the distinction between parental cognition and behavior was not always clear in previous theories and research. Though we excluded such constructs from the analysis that are traditionally referred to in the literature as parenting practices, parenting styles, or parenting behaviors, we are aware of the fact that these concepts also include a cognitive component. Furthermore, the categorization of parental cognitions as stable or situational is somewhat arbitrary (106), they are more likely two endpoints of the single continuum than distinct categories, and therefore these characteristics could be better treated dimensionally. Similarly, because parenting involves an interaction between the parent and the child, it was sometimes debatable whether the reference of the cognition was the parent, the child, or the interaction *per se*.

#### 4.2.4 The low number of independent contrasts

Because of the low number of independent contrasts in most moderators, we decided to report preliminary descriptive results of subgroup analyzes, i.e., the average effects in the different categories without statistically contrasting them. With more cumulating evidence these issues should be revisited.

Only one study included pregnant women (78), and a single study used a sample of mothers of infants (79). Even in this early period, maternal ADHD symptoms were associated with more negative expectations about the parental role and lower parental self-efficacy. These results suggest that parent characteristics could influence parental cognitions beyond child-driven effects. On the other hand, they draw attention to the importance of early prevention programs in mothers living with ADHD. More research is needed in this field.

Only a single study reported the relationships between parental cognitions and symptoms of inattention and hyperactivity/impulsivity separately, and all other studies used the total score of the rating scales as a general measure of ADHD symptoms. However, previous research found different associations of attention deficit and hyperactivity/impulsivity factors with comorbid conditions, cognitive variables, and different domains of functional impairment (131). Therefore, further research should explore the impact of different symptom domains on parental cognitions.

The interpersonal problems associated with adult ADHD are not limited to the parent–child relationship but can also affect cooperation between parents (74). Although a recent meta-analysis found that coparenting was associated with child mental well-being (132), only one study has examined the relationship between parental ADHD and coparenting. Further studies are needed in this area.

#### 4.2.5 Cross-sectional data

We analyzed cross-sectional data which did not allow us to test cause-effect relationships. Longitudinal studies are needed to uncover the possible bidirectional nature of parental ADHD symptom-level and dysfunctional cognitions.

## 5 Conclusion

Despite these limitations, the present study contributed to the research on parental cognitions by giving insight into the strength of the association between parental ADHD symptoms and parental cognitions. Though the analysis might be impacted by publication bias, our results suggest a significant association of small effect size between ADHD symptom levels and dysfunctional parental cognitions. Dysfunctional parental cognitions may play a central mediator role between parental ADHD and parent and child outcomes. Considering the high heritability of ADHD (133), and the huge amount of evidence on its familiar risk factors (134, 135), targeting parental cognitions in parent training programs is warranted.

## Data availability statement

The datasets presented in this study can be found in online repositories. The names of the repository/repositories and accession number(s) can be found at: OSF https://osf.io/pnur7/?view_only=181ede69724a4c3e8736cedac9d1ccc2.

## Author contributions

MM: Conceptualization, Formal analysis, Funding acquisition, Investigation, Methodology, Project administration, Writing – original draft, Writing – review & editing. BK: Conceptualization, Formal analysis, Methodology, Writing – review & editing. JJ: Data curation, Formal analysis, Investigation, Writing – review & editing. FL: Data curation, Formal analysis, Investigation, Writing – review & editing. RK: Formal analysis, Methodology, Supervision, Writing – review & editing.

## Footnotes

^1^ https://osf.io/pnur7/?view_only=181ede69724a4c3e8736cedac9d1ccc2

## References

[1] American Psychiatric Association. Diagnostic and statistical manual of mental disorders: DSM-5, vol. 5. Washington, DC: American Psychiatric Association (2013).

[2] Sibley MH Mitchell JT Becker SP. Method of adult diagnosis influences estimated persistence of childhood ADHD: a systematic review of longitudinal studies. Lancet Psychiatry. (2016) 3:1157–65. doi: 10.1016/S2215-0366(16)30190-0, PMID: 27745869

[3] Song P Zha M Yang Q Zhang Y Li X Rudan I. The prevalence of adult attention-deficit hyperactivity disorder: a global systematic review and meta-analysis. J Glob Health. (2022) 11:04009. doi: 10.7189/jogh.11.04009 PMC7916320 33692893

[4] Fayyad J Sampson NA Hwang I Adamowski T Aguilar-Gaxiola S Al-Hamzawi A . The descriptive epidemiology of DSM-IV adult ADHD in the World Health Organization world mental health surveys. Atten Deficit Hyperact Disord. (2017) 9:47–65. doi: 10.1007/s12402-016-0208-3, PMID: 27866355 PMC5325787

[5] Biederman J. Impact of comorbidity in adults with attention-deficit/hyperactivity disorder. J Clin Psychiatry. (2004) 65:3–7. PMID: 15046528

[6] McGough JJ Smalley SL McCracken JT Yang M Del’Homme M Lynn DE . Psychiatric comorbidity in adult attention deficit hyperactivity disorder: findings from multiplex families. Am J Psychiatry. (2005) 162:1621–7. doi: 10.1176/appi.ajp.162.9.1621, PMID: 16135620

[7] Schiweck C Arteaga-Henriquez G Aichholzer M Thanarajah SE Vargas-Cáceres S Matura S . Comorbidity of ADHD and adult bipolar disorder: a systematic review and meta-analysis. Neurosci Biobehav Rev. (2021) 124:100–23. doi: 10.1016/j.neubiorev.2021.01.017, PMID: 33515607

[8] Oliva F Mangiapane C Nibbio G Berchialla P Colombi N Vigna-Taglianti FD. Prevalence of cocaine use and cocaine use disorder among adult patients with attention-deficit/hyperactivity disorder: a systematic review and meta-analysis. J Psychiatr Res. (2021) 143:587–98. doi: 10.1016/j.jpsychires.2020.11.021, PMID: 33199055

[9] Luderer M Ramos Quiroga JA Faraone SV Zhang-James Y Reif A. Alcohol use disorders and ADHD. Neurosci Biobehav Rev. (2021) 128:648–60. doi: 10.1016/j.neubiorev.2021.07.010 34265320

[10] Capusan AJ Bendtsen P Marteinsdottir I Larsson H. Comorbidity of adult ADHD and its subtypes with substance use disorder in a large population-based epidemiological study. J Atten Disord. (2019) 23:1416–26. doi: 10.1177/1087054715626511, PMID: 26838558

[11] El Archi S Barrault S Brunault P Ribadier A Varescon I. Co-occurrence of adult ADHD symptoms and problematic internet use and its links with impulsivity, emotion regulation, anxiety, and depression. Front Psychiatry. (2022) 13:792206. doi: 10.3389/fpsyt.2022.792206 35492700 PMC9045584

[12] Fadeuilhe C Daigre C Richarte V Grau-López L Palma-Álvarez RF Corrales M . Insomnia disorder in adult attention-deficit/hyperactivity disorder patients: clinical, comorbidity, and treatment correlates. Front Psychiatry. (2021) 12:3889. doi: 10.3389/fpsyt.2021.663889, PMID: 34122179 PMC8187558

[13] Matthies S Philipsen A. Comorbidity of personality disorders and adult attention deficit hyperactivity disorder (ADHD)–review of recent findings. Curr Psychiatry Rep. (2016) 18:33. doi: 10.1007/s11920-016-0675-4 26893231

[14] Beheshti A Chavanon ML Christiansen H. Emotion dysregulation in adults with attention deficit hyperactivity disorder: a meta-analysis. BMC Psychiatry. (2020) 20:120. doi: 10.1186/s12888-020-2442-7 32164655 PMC7069054

[15] Boonstra AM Oosterlaan J Sergeant JA Buitelaar JK. Executive functioning in adult ADHD: a meta-analytic review. Psychol Med. (2005) 35:1097–108. doi: 10.1017/S003329170500499X, PMID: 16116936

[16] Mowinckel AM Pedersen ML Eilertsen E Biele G. A meta-analysis of decision-making and attention in adults with ADHD. J Atten Disord. (2015) 19:355–67. doi: 10.1177/1087054714558872, PMID: 25477020

[17] Bodalski EA Knouse LE Kovalev D. Adult ADHD, emotion dysregulation, and functional outcomes: examining the role of emotion regulation strategies. J Psychopathol Behav Assess. (2019) 41:81–92. doi: 10.1007/s10862-018-9695-1

[18] Quintero J Morales I Vera R Zuluaga P Fernández A. The impact of adult ADHD in the quality of life profile. J Atten Disord. (2019) 23:1007–16. doi: 10.1177/1087054717733046 28974134

[19] Ginapp CM Greenberg NR Macdonald-Gagnon G Angarita GA Bold KW Potenza MN. The experiences of adults with ADHD in interpersonal relationships and online communities: a qualitative study. SSM Qual Res Health. (2023) 3:100223. doi: 10.1016/j.ssmqr.2023.100223, PMID: 37539360 PMC10399076

[20] Ryan J Ross S Reyes R Kosmerly S Rogers M. Social functioning among college students diagnosed with ADHD and the mediating role of emotion regulation. Emot Behav Diffic. (2016) 21:1–16. doi: 10.1080/13632752.2016.1235329

[21] Ginapp CM Macdonald-Gagnon G Angarita GA Bold KW Potenza MN. The lived experiences of adults with attention-deficit/hyperactivity disorder: a rapid review of qualitative evidence. Front Psychiatry. (2022) 13:9321. doi: 10.3389/fpsyt.2022.949321, PMID: 36032220 PMC9403235

[22] Chronis-Tuscano A Raggi VL Clarke TL Rooney ME Diaz Y Pian J. Associations between maternal attention-deficit/hyperactivity disorder symptoms and parenting. J Abnorm Child Psychol. (2008) 36:1237–50. doi: 10.1007/s10802-008-9246-4, PMID: 18553132 PMC3715319

[23] Park JL Johnston C. Parental ADHD symptoms and parenting behaviors. ADHD Rep. (2019) 27:1–7. doi: 10.1521/adhd.2019.27.3.1 28601690

[24] Theule J Wiener J Rogers MA Marton I. Predicting parenting stress in families of children with ADHD: parent and contextual factors. J Child Fam Stud. (2011) 20:640–7. doi: 10.1007/s10826-010-9439-7

[25] Wang CH Mazursky-Horowitz H Chronis-Tuscano A. Delivering evidence-based treatments for child attention-deficit/hyperactivity disorder (ADHD) in the context of parental ADHD. Curr Psychiatry Rep. (2014) 16:474. doi: 10.1007/s11920-014-0474-8, PMID: 25135774 PMC4664577

[26] Chronis-Tuscano A Wang CH Woods KE Strickland J Stein MA. Parent ADHD and evidence-based treatment for their children: review and directions for future research. J Abnorm Child Psychol. (2017) 45:501–17. doi: 10.1007/s10802-016-0238-5, PMID: 28025755 PMC5357146

[27] Johnston C Mash EJ Miller N Ninowski JE. Parenting in adults with attention-deficit/hyperactivity disorder (ADHD). Clin Psychol Rev. (2012) 32:215–28. doi: 10.1016/j.cpr.2012.01.007, PMID: 22459785 PMC4838457

[28] Sanders MR Mazzucchelli TG. The promotion of self-regulation through parenting interventions. Clin Child Fam Psychol Rev. (2013) 16:1–17. doi: 10.1007/s10567-013-0129-z 23397366

[29] Calam RM Bee PE. Self-regulation and parental mental health In: Sanders MR Morawska A, editors. Handbook of parenting and child development across the lifespan. Cham: Springer International Publishing (2018). 371–94.

[30] Colalillo S. *Associations between maternal executive functions and parenting behavior: Are they moderated by parental childrearing attitudes?* [PhD Thesis]. Columbia: University of British Columbia (2018).

[31] Diercks CM Gunther KE Teti DM Lunkenheimer E. Ecological validity in measuring parents’ executive function. Child Dev Perspect. (2022) 16:208–14. doi: 10.1111/cdep.12464, PMID: 36590076 PMC9799100

[32] Tomlinson RC Hyde LW Weigard AS Klump KL Burt SA. The role of parenting in the intergenerational transmission of executive functioning: a genetically informed approach. Dev Psychopathol. (2022) 34:1731–43. doi: 10.1017/S0954579422000645, PMID: 35957575 PMC9922338

[33] Crouch JL McKay ER Lelakowska G Hiraoka R Rutledge E Bridgett DJ . Do emotion regulation difficulties explain the association between executive functions and child physical abuse risk? Child Abuse Negl. (2018) 80:99–107. doi: 10.1016/j.chiabu.2018.03.003, PMID: 29587198

[34] Zaidman-Zait A Shilo I. Parental ADHD symptoms and inhibitory control in relation to parenting among mothers of children with and without ADHD. J Atten Disord. (2021) 25:389–402. doi: 10.1177/1087054718808063, PMID: 30442044

[35] Newark PE Stieglitz RD. Therapy-relevant factors in adult ADHD from a cognitive behavioural perspective. Atten Deficit Hyperact Disord. (2010) 2:59–72. doi: 10.1007/s12402-010-0023-1, PMID: 21432591

[36] Ramsay JR. Cognitive behavior therapy model of adult ADHD In: Ramsay JR, editor. Rethinking adult ADHD: Helping clients turn intentions into actions. Washington, DC: American Psychological Association (2020). 41–61.

[37] Newark PE Elsaesser M Stieglitz RD. Self-esteem, self-efficacy, and resources in adults with ADHD. J Atten Disord. (2016) 20:279–90. doi: 10.1177/1087054712459561, PMID: 23074301

[38] Cook J Knight E Hume I Qureshi A. The self-esteem of adults diagnosed with attention-deficit/hyperactivity disorder (ADHD): a systematic review of the literature. ADHD Atten Deficit Hyperact Disord. (2014) 6:249–68. doi: 10.1007/s12402-014-0133-2, PMID: 24668198

[39] Harpin V Mazzone L Raynaud JP Kahle J Hodgkins P. Long-term outcomes of ADHD: a systematic review of self-esteem and social function. J Atten Disord. (2016) 20:295–305. doi: 10.1177/1087054713486516 23698916

[40] Lücke C Lam AP Muller HHO Philipsen A. New psychotherapeutic approaches in adult ADHD - acknowledging biographical factors. J Neurol Neuromed. (2017) 7:1. doi: 10.29245/2572.942X/2017/7.1138

[41] Máté O Somogyi K Miklósi M. Cognitive conceptualization of adult attention deficit hyperactivity disorder: a systematic review. Psychiatr Hung. (2015) 30:68–77. PMID: 25867890

[42] Knouse LE Mitchell JT. Incautiously optimistic: positively Valenced cognitive avoidance in adult ADHD. Cogn Behav Pract. (2015) 22:192–202. doi: 10.1016/j.cbpra.2014.06.003, PMID: 25908901 PMC4403795

[43] Lui JH Johnston C Lee CM Lee-Flynn SC. Parental ADHD symptoms and self-reports of positive parenting. J Consult Clin Psychol. (2013) 81:988–98. doi: 10.1037/a0033490 23796318

[44] Page MJ McKenzie JE Bossuyt PM Boutron I Hoffmann TC Mulrow CD . The PRISMA 2020 statement: an updated guideline for reporting systematic reviews. Int J Surg. (2021) 88:105906. doi: 10.1016/j.ijsu.2021.105906, PMID: 33789826

[45] American Psychiatric Association. Dsm-iv-Tr. Diagnostic and Statistical Manual of Mental Disorders. Virginia, US: American Psychiatric Association (2000).

[46] Deater-Deckard K. Parenting stress. New Haven: Yale University Press (2008).

[47] Evans N Lasen M Tsey K. Appendix a: effective public health practice project (EPHPP) quality assessment tool for quantitative studies. Syst Rev Rural Dev Res Charact Des Qual Engagem Sustain U S A. (2015) 2015:45–63.

[48] Borenstein M Hedges LV Higgins JPT Rothstein HR. Introduction to Meta-analysis. New York: John Wiley & Sons (2021). 547 p.

[49] Rosenthal R. The file drawer problem and tolerance for null results. Psychol Bull. (1979) 86:638–41. doi: 10.1037/0033-2909.86.3.638

[50] Butcher JL Niec LN. Mothers’ attributions about child misbehavior: can situational suggestions change general perceptions? CHILD Fam Behav Ther. (2017) 39:131–47. doi: 10.1080/07317107.2017.1307680

[51] Cappe E Bolduc M Rouge M Saiag M Delorme R. Quality of life, psychological characteristics, and adjustment in parents of children with attention-deficit/hyperactivity disorder. Qual Life Res. (2017) 26:1283–94. doi: 10.1007/s11136-016-1446-8, PMID: 27798755

[52] Chacko A Wymbs BT Rajwan E Wymbs F Feirsen N. Characteristics of parents of children with ADHD who never attend, drop out, and complete behavioral parent training. J Child Fam Stud. (2017) 26:950–60. doi: 10.1007/s10826-016-0618-z

[53] Chronis A Gamble S Roberts J Pelham W. Cognitive-behavioral depression treatment for mothers of children with attention-deficit/hyperactivity disorder. Behav Ther. (2006) 37:143–58. doi: 10.1016/j.beth.2005.08.001, PMID: 16942968

[54] Feinfield K Baker B. Empirical support for a treatment program for families of young children with externalizing problems. J Clin Child Adolesc Psychol. (2004) 33:182–95. doi: 10.1207/S15374424JCCP3301_17, PMID: 15028552

[55] Hautmann C Hoijtink H Eichelberger I Hanisch C Pluck J Walter D . One-year follow-up of a parent management training for children with externalizing behaviour problems in the real world. Behav Cogn Psychother. (2009) 37:379–96. doi: 10.1017/S135246580999021X, PMID: 19619384

[56] Hautmann C Eichelberger I Hanisch C Pluck J Walter D Dopfner M. The severely impaired do profit most: short-term and long-term predictors of therapeutic change for a parent management training under routine care conditions for children with externalizing problem behavior. Eur Child Adolesc Psychiatry. (2010) 19:419–30. doi: 10.1007/s00787-009-0072-1 19915886

[57] Heath C Curtis D Fan W McPherson R. The association between parenting stress, parenting self-efficacy, and the clinical significance of child ADHD symptom change following behavior therapy. Child Psychiatry Hum Dev. (2015) 46:118–29. doi: 10.1007/s10578-014-0458-2, PMID: 24668566

[58] Park J Johnston C Colalillo S Williamson D. Parents’ attributions for negative and positive child behavior in relation to parenting and child problems. J Clin Child Adolesc Psychol. (2018) 47:S63–75. doi: 10.1080/15374416.2016.1144191, PMID: 27070717

[59] Van der Zanden RAP Speetjens PAM Arntz KSE Onrust SA. Online group course for parents with mental illness: development and pilot study. J Med Internet Res. (2010) 12:1394. doi: 10.2196/jmir.1394 PMC3057319 21169178

[60] Vural P Akkaya C Kucukparlak I Ercan I Eracar N. Psychodramatic group psychotherapy as a parental intervention in attention deficit hyperactivity disorder: a preliminary study. Arts Psychother. (2014) 41:233–9. doi: 10.1016/j.aip.2014.02.004

[61] Noordermeer S Luman M Weeda W Buitelaar J Richards J Hartman C . Risk factors for comorbid oppositional defiant disorder in attention-deficit/hyperactivity disorder. Eur Child Adolesc Psychiatry. (2017) 26:1155–64. doi: 10.1007/s00787-017-0972-4, PMID: 28283834 PMC5610221

[62] Van den Hoofdakker B Hoekstra P Van der Veen-Mulders L Sytema S Emmelkamp P Minderaa R . Paternal influences on treatment outcome of behavioral parent training in children with attention-deficit/hyperactivity disorder. Eur Child Adolesc Psychiatry. (2014) 23:1071–9. doi: 10.1007/s00787-014-0557-4, PMID: 24878676

[63] Kroeger RA. Parental happiness and strain among young adult parents diagnosed with attention deficit hyperactivity disorder. Chronic Illn. (2018) 14:69–75. doi: 10.1177/1742395317694701, PMID: 29226701

[64] Moen OL Hedelin B Hal-Lord ML. Parental perception of family functioning in everyday life with a child with ADHD. Scand J Public Health. (2015) 43:10–7. doi: 10.1177/1403494814559803, PMID: 25420708

[65] Williamson D Johnston C Noyes A Stewart K Weiss MD. Attention-deficit/hyperactivity disorder symptoms in mothers and fathers: family level interactions in relation to parenting. J Abnorm Child Psychol. (2017) 45:485–500. doi: 10.1007/s10802-016-0235-8, PMID: 27909931

[66] Johnston C Williamson D Noyes A Stewart K Weiss MD. Parent and child ADHD symptoms in relation to parental attitudes and parenting: testing the similarity-fit hypothesis. J Clin Child Adolesc Psychol. (2018) 47:S127–36. doi: 10.1080/15374416.2016.1169538, PMID: 27359250

[67] Haack LM Jiang Y Delucchi K Kaiser N McBurnett K Hinshaw S . Parental cognitive errors mediate parental psychopathology and ratings of child inattention. Fam Process. (2017) 56:716–33. doi: 10.1111/famp.12252, PMID: 27663189 PMC5365376

[68] Fabrikant-Abzug G Friedman L Pfiffner L. Examining relations between parent and child psychopathology in children with ADHD: do parent cognitions matter? J Psychopathol Behav Assess. (2023) 45:75–87. doi: 10.1007/s10862-023-10023-1

[69] Watkins SJ. *The relationship between symptoms of attention-deficit/hyperactivity disorder and self-reported parental cognitions and behaviours in mothers of young infants* [Master’s Thesis]. University of Calgary (2006).

[70] Williamson DK. Maternal ADHD symptoms and parenting stress: the roles of personality and parenting self-efficacy beliefs [Dissertation]. University of British Columbia (2016).

[71] Lowry LS Schatz NK Fabiano GA. Exploring parent beliefs and behavior: the contribution of ADHD symptomology within mothers and fathers. J Atten Disord. (2018) 22:1255–65. doi: 10.1177/1087054714562587 25555630

[72] Psychogiou L Daley D Thompson M Sonuga-Barke E. Parenting empathy: associations with dimensions of parent and child psychopathology. Br J Dev Psychol. (2008) 26:221–32. doi: 10.1348/02615100X238582

[73] Richards J Vasquez A Rommelse N Oosterlaan J Hoekstra P Franke B . A follow-up study of maternal expressed emotion toward children with attention-deficit/hyperactivity disorder (ADHD): relation with severity and persistence of ADHD and comorbidity. J Am Acad Child Adolesc Psychiatry. (2014) 53:311–319.e1. doi: 10.1016/j.jaac.2013.11.011, PMID: 24565358 PMC4066112

[74] Williamson D Johnston C. Marital and coparenting relationships: associations with parent and child symptoms of ADHD. J Atten Disord. (2016) 20:684–94. doi: 10.1177/1087054712471717 23390081

[75] Park JL Johnston C. Mothers’ attributions for positive and negative child behavior: associations with mothers’ ADHD symptoms. J Atten Disord. (2019) 23:475–86. doi: 10.1177/1087054716669590 27650394

[76] Williamson D Johnston C. Maternal ADHD symptoms and parenting stress: the roles of parenting self-efficacy beliefs and neuroticism. J Atten Disord. (2019) 23:493–505. doi: 10.1177/1087054717693373, PMID: 28201945

[77] Banks T Ninowski JE Mash EJ Semple DL. Parenting behavior and cognitions in a community sample of mothers with and without symptoms of attention-deficit/hyperactivity disorder. J Child Fam Stud. (2008) 17:28–43. doi: 10.1007/s10826-007-9139-0

[78] Ninowski JE Mash EJ Benzies KM. Symptoms of attention-deficit/hyperactivity disorder in first-time expectant women: relations with parenting cognitions and behaviors. Infant Ment Health J. (2007) 28:54–75. doi: 10.1002/imhj.20122, PMID: 28640382

[79] Psychogiou L Daley DM Thompson MJ Sonuga-Barke EJ. Mothers’ expressed emotion toward their school-aged sons: associations with child and maternal symptoms of psychopathology. Eur Child Adolesc Psychiatry. (2007) 16:458–64. doi: 10.1007/s00787-007-0619-y, PMID: 17876512

[80] Watkins SJ Mash EJ. Sub-clinical levels of symptoms of attention-deficit/hyperactivity disorder and self-reported parental cognitions and behaviours in mothers of young infants. J Reprod Infant Psychol. (2009) 27:70–88. doi: 10.1080/02646830801918448

[81] Moroney E Tung I Brammer WA Peris TS Lee SS. Externalizing outcomes of youth with and without ADHD: time-varying prediction by parental ADHD and mediated effects. J Abnorm Child Psychol. (2017) 45:457–70. doi: 10.1007/s10802-016-0215-z, PMID: 27796692

[82] Lindstrom T Suttner A Forster M Bolte S Hirvikoski T. Is parents’ ADHD symptomatology associated with the clinical feasibility or effectiveness of a psychoeducational program targeting their Children’s ADHD? J Atten Disord. (2022) 26:1653–67. doi: 10.1177/10870547221092120, PMID: 35491992 PMC9373197

[83] Sonuga-Barke EJ Daley D Thompson M. Does maternal ADHD reduce the effectiveness of parent training for preschool children’s ADHD? J Am Acad Child Adolesc Psychiatry. (2002) 41:696–702. doi: 10.1097/00004583-200206000-00009 12049444

[84] Barkley RA Murphy KR. Attention-deficit hyperactivity disorder: A clinical workbook. New York: Guilford Press (2006).

[85] DuPaul GJ Power TJ Anastopoulos AD Reid R. ADHD rating scale–IV: Checklists, norms, and clinical interpretation. New York: Guilford press (1998).

[86] Barkley RA. Barkley adult ADHD rating scale-IV (BAARS-IV). New York: Guilford Press (2011).

[87] Conners CK Erhardt D Sparrow MA. Conners ‘adult ADHD rating scales, technical manual. New York: Multihealth Systems Inc. (1999).

[88] Kessler RC Adler LA Gruber MJ Sarawate CA Spencer T Van Brunt DL. Validity of the World Health Organization adult ADHD self-report scale (ASRS) screener in a representative sample of health plan members. Int J Methods Psychiatr Res. (2007) 16:52–65. doi: 10.1002/mpr.208, PMID: 17623385 PMC2044504

[89] Achenbach TM Rescorla LA. Manual for the ASEBA adult forms and profiles. Burlington, VT: University of Vermont, Research Center for Children, Youth (2003).

[90] Coleman PK Karraker KH. Parenting self-efficacy among mothers of school-age children: conceptualization, measurement, and correlates. Fam Relat. (2000) 49:13–24. doi: 10.1111/j.1741-3729.2000.00013.x

[91] Johnston C Mash EJ. A measure of parenting satisfaction and efficacy. J Clin Child Psychol. (1989) 18:167–75. doi: 10.1207/s15374424jccp1802_8

[92] Johnston C Ohan JL. The importance of parental attributions in families of children with attention-deficit/hyperactivity and disruptive behavior disorders. Clin Child Fam Psychol Rev. (2005) 8:167–82. doi: 10.1007/s10567-005-6663-6, PMID: 16151616

[93] Boivin M Pérusse D Dionne G Saysset V Zoccolillo M Tarabulsy GM . The genetic-environmental etiology of parents’ perceptions and self-assessed behaviours toward their 5-month-old infants in a large twin and singleton sample. J Child Psychol Psychiatry. (2005) 46:612–30. doi: 10.1111/j.1469-7610.2004.00375.x, PMID: 15877767

[94] Harty M. The validation of a task-specific measure of parenting self-efficacy for use with mothers of young children. Pretoria: University of Pretoria (2009).

[95] Campis LK Lyman RD Prentice-Dunn S. The parental locus of control scale: development and validation. J Clin Child Psychol. (1986) 15:260–7. doi: 10.1207/s15374424jccp1503_10

[96] Brannan AM Heflinger CA Bickman L. The caregiver strain questionnaire: measuring the impact on the family of living with a child with serious emotional disturbance. J Emot Behav Disord. (1997) 5:212–22. doi: 10.1177/106342669700500404

[97] Coleman P Nelson ES Sundre DL. The relationship between prenatal expectations and postnatal attitudes among first-time mothers. J Reprod Infant Psychol. (1999) 17:27–39. doi: 10.1080/02646839908404582

[98] Johnston C Freeman W. Attributions for child behavior in parents of children without behavior disorders and children with attention deficit-hyperactivity disorder. J Consult Clin Psychol. (1997) 65:636–45. doi: 10.1037/0022-006X.65.4.636 9256565

[99] Kaiser NM Hinshaw SP Pfiffner LJ. Parent cognitions and behavioral parent training: engagement and outcomes. ADHD Rep. (2010) 18:6–12. doi: 10.1521/adhd.2010.18.1.6

[100] Brestan EV Eyberg SM Algina J Johnson SB Boggs SR. How annoying is it? Defining parental tolerance for child misbehavior. Child Fam Behav Ther. (2003) 25:1–15. doi: 10.1300/J019v25n02_01

[101] Davis MH. The effects of dispositional empathy on emotional reactions and helping: a multidimensional approach. J Pers. (1983) 51:167–84. doi: 10.1111/j.1467-6494.1983.tb00860.x

[102] Geller J Johnston C. Predictors of mothers’ responses to child noncompliance: attributions and attitudes. J Clin Child Psychol. (1995) 24:272–8. doi: 10.1207/s15374424jccp2403_4

[103] Magaña AB Goldstein MJ Karno M Miklowitz DJ Jenkins J Falloon IRH. A brief method for assessing expressed emotion in relatives of psychiatric patients. Psychiatry Res. (1986) 17:203–12. doi: 10.1016/0165-1781(86)90049-1 3704028

[104] Brown GW Rutter M. The measurement of family activities and relationships: a methodological study. Hum Relat. (1966) 19:241–63. doi: 10.1177/001872676601900301

[105] Konold TR Abidin RR. Parenting Alliance: a multifactor perspective. Assessment. (2001) 8:47–65. doi: 10.1177/107319110100800105, PMID: 11310726

[106] Johnston C Park JL Miller NV. Parental cognitions: relations to parenting and child behavior. Handb Parent Child Dev Lifesp. (2018) 1:395–414. doi: 10.1007/978-3-319-94598-9_17

[107] Miklosi M Mate O Somogyi K Szabo M. Adult attention deficit hyperactivity disorder symptoms, perceived stress, and well-being the role of early maladaptive schemata. J Nerv Ment Dis. (2016) 204:364–9. doi: 10.1097/NMD.0000000000000472 26825377

[108] Philipsen A Lam AP Breit S Lücke C Müller HH Matthies S. Early maladaptive schemas in adult patients with attention deficit hyperactivity disorder. ADHD Atten Deficit Hyperact Disord. (2017) 9:101–11. doi: 10.1007/s12402-016-0211-8, PMID: 28012033

[109] Pan MR Zhang SY Chen CL Qiu SW Liu L Li HM . Bidirectional associations between maladaptive cognitions and emotional symptoms, and their mediating role on the quality of life in adults with ADHD: a mediation model. Front Psychiatry. (2023) 14:522. doi: 10.3389/fpsyt.2023.1200522, PMID: 37547201 PMC10400449

[110] Knouse LE Mitchell JT Kimbrel NA Anastopoulos AD. Development and evaluation of the ADHD cognitions scale for adults. J Atten Disord. (2019) 23:1090–100. doi: 10.1177/1087054717707580, PMID: 28528555 PMC5665714

[111] Avinun R Knafo A. Parenting as a reaction evoked by children’s genotype: a meta-analysis of children-as-twins studies. Pers Soc Psychol Rev. (2014) 18:87–102. doi: 10.1177/1088868313498308 23940232

[112] Yan N Ansari A Peng P. Reconsidering the relation between parental functioning and child externalizing behaviors: a meta-analysis on child-driven effects. J Fam Psychol. (2021) 35:225–35. doi: 10.1037/fam0000805, PMID: 33104378

[113] De la Paz L Mooney MA Ryabinin P Neighbor C Antovich D Nigg JT . Youth polygenic scores, youth ADHD symptoms, and parenting dimensions: an evocative gene-environment correlation study. Res Child Adolesc Psychopathol. (2023) 51:665–77. doi: 10.1007/s10802-023-01024-5, PMID: 36645612 PMC10560546

[114] Leerkes EM Crockenberg SC. The development of maternal self-efficacy and its impact on maternal behavior. Infancy. (2002) 3:227–47. doi: 10.1207/S15327078IN0302_7 33451204

[115] Jones TL Prinz RJ. Potential roles of parental self-efficacy in parent and child adjustment: a review. Clin Psychol Rev. (2005) 25:341–63. doi: 10.1016/j.cpr.2004.12.004, PMID: 15792853

[116] Albanese AM Russo GR Geller PA. The role of parental self-efficacy in parent and child well-being: a systematic review of associated outcomes. Child Care Health Dev. (2019) 45:333–63. doi: 10.1111/cch.12661 30870584

[117] Jiang Y Haack LM Delucchi K Rooney M Hinshaw SP McBurnett K . Improved parent cognitions relate to immediate and follow-up treatment outcomes for children with ADHD-predominantly inattentive presentation. Behav Ther. (2018) 49:567–79. doi: 10.1016/j.beth.2017.11.007, PMID: 29937258 PMC6020154

[118] Ragozin AS Basham RB Crnic KA Greenberg MT Robinson NM. Effects of maternal age on parenting role. Dev Psychol. (1982) 18:627–34. doi: 10.1037/0012-1649.18.4.627

[119] Fang Y Boelens M Windhorst DA Raat H van Grieken A. Factors associated with parenting self-efficacy: a systematic review. J Adv Nurs. (2021) 77:2641–61. doi: 10.1111/jan.14767, PMID: 33590585 PMC8248335

[120] Faraone SV Biederman J Mick E. The age-dependent decline of attention deficit hyperactivity disorder: a meta-analysis of follow-up studies. Psychol Med. (2006) 36:159–65. doi: 10.1017/S003329170500471X, PMID: 16420712

[121] Attoe DE Climie EA. Miss. Diagnosis: a systematic review of ADHD in adult women. J Atten Disord. (2023) 27:645–57. doi: 10.1177/10870547231161533 36995125 PMC10173330

[122] Williamson D Johnston C. Gender differences in adults with attention-deficit/hyperactivity disorder: a narrative review. Clin Psychol Rev. (2015) 40:15–27. doi: 10.1016/j.cpr.2015.05.005, PMID: 26046624

[123] Young S Adamo N Ásgeirsdóttir BB Branney P Beckett M Colley W . Females with ADHD: an expert consensus statement taking a lifespan approach providing guidance for the identification and treatment of attention-deficit/ hyperactivity disorder in girls and women. BMC Psychiatry. (2020) 20:404. doi: 10.1186/s12888-020-02707-9, PMID: 32787804 PMC7422602

[124] Psychogiou L Daley D Thompson M Sonuga-Barke E. Testing the interactive effect of parent and child ADHD on parenting in mothers and fathers: a further test of the similarity-fit hypothesis. Br J Dev Psychol. (2007) 25:419–33. doi: 10.1348/026151006X170281

[125] Slobodin O Davidovitch M. Gender differences in objective and subjective measures of ADHD among clinic-referred children. Front Hum Neurosci. (2019) 13:441. doi: 10.3389/fnhum.2019.00441, PMID: 31920599 PMC6923191

[126] Derks EM Dolan CV Hudziak JJ Neale MC Boomsma DI. Assessment and etiology of attention deficit hyperactivity disorder and oppositional defiant disorder in boys and girls. Behav Genet. (2007) 37:559–66. doi: 10.1007/s10519-007-9153-4, PMID: 17443404 PMC1914288

[127] Klefsjö U Kantzer AK Gillberg C Billstedt E. The road to diagnosis and treatment in girls and boys with ADHD – gender differences in the diagnostic process. Nord J Psychiatry. (2021) 75:301–5. doi: 10.1080/08039488.2020.1850859, PMID: 33241961

[128] Mowlem F Agnew-Blais J Taylor E Asherson P. Do different factors influence whether girls versus boys meet ADHD diagnostic criteria? Sex differences among children with high ADHD symptoms. Psychiatry Res. (2019) 272:765–73. doi: 10.1016/j.psychres.2018.12.128, PMID: 30832197 PMC6401208

[129] Pipe A Ravindran N Paric A Patterson B Van Ameringen M Ravindran AV. Treatments for child and adolescent attention deficit hyperactivity disorder in low and middle-income countries: a narrative review. Asian J Psychiatry. (2022) 76:103232. doi: 10.1016/j.ajp.2022.103232, PMID: 35987096

[130] Toplak ME Sorge GB Flora DB Chen W Banaschewski T Buitelaar J . The hierarchical factor model of ADHD: invariant across age and national groupings? J Child Psychol Psychiatry. (2012) 53:292–303. doi: 10.1111/j.1469-7610.2011.02500.x, PMID: 22084976 PMC3272099

[131] Willcutt EG. The prevalence of DSM-IV attention-deficit/hyperactivity disorder: a Meta-analytic review. Neurotherapeutics. (2012) 9:490–9. doi: 10.1007/s13311-012-0135-8, PMID: 22976615 PMC3441936

[132] Zhao F Wu H Li Y Zhang H Hou J. The association between Coparenting behavior and internalizing/externalizing problems of children and adolescents: a Meta-analysis. Int J Environ Res Public Health. (2022) 19:10346. doi: 10.3390/ijerph191610346, PMID: 36011980 PMC9407961

[133] Larsson H Chang Z D’Onofrio BM Lichtenstein P. The heritability of clinically diagnosed attention deficit hyperactivity disorder across the lifespan. Psychol Med. (2014) 44:2223–9. doi: 10.1017/S0033291713002493, PMID: 24107258 PMC4071160

[134] Claussen AH Holbrook JR Hutchins HJ Robinson LR Bloomfield J Meng L . All in the family? A systematic review and Meta-analysis of parenting and family environment as risk factors for attention-deficit/hyperactivity disorder (ADHD) in children. Prev Sci. (2022) 1:1–23. doi: 10.1007/s11121-022-01358-4, PMID: 35438451 PMC9017071

[135] Mazzeschi C Buratta L Germani A Cavallina C Ghignoni R Margheriti M . Parental reflective functioning in mothers and fathers of children with ADHD: issues regarding assessment and implications for intervention. Front Public Health. (2019) 7:7. doi: 10.3389/fpubh.2019.00263 31572704 PMC6753962

