# Supplementary material for: Adult attention-deficit/hyperactivity symptoms and parental cognitions: a meta-analysis
Source: Front Psychiatry. 2024 Jan 9;14:1321078. doi: 10.3389/fpsyt.2023.1321078 (PMC10807045; doi:10.3389/fpsyt.2023.1321078)
Supplement: Supplementary file 1 [file Table_1.DOCX]

Supplementary Material

# Table S1

Final search term

| ADHD | "attention-deficit/hyperactivity disorder" OR "attention deficit disorder with hyperactivity" OR "attention deficit disorder" OR "hyperkinetic disorder" OR "hyperactivity disorder" OR "attention deficit hyperactivity disorder" OR “ADHD” OR “adhd” OR "attention deficit" OR “attention-deficit” OR “attention-deficit/hyperactivity” OR “hyperactiv*” OR “hyperkine*” OR “inattent* |
| --- | --- |
| AND |  |
| Parental cognitions | "parent* attitude*" OR "parent* attribution*" OR "parent* perception*" OR "parent* expectation*" OR "parent* belief*" OR "parent* cognition*" OR "parent* schema*" OR “parent* criticism” OR “parent* self-efficacy” OR “parent* self-esteem” OR “parent* locus of control” OR “parent* empathy” OR “parental role satisfaction” OR "mother* attitude*" OR "mother* attribution*" OR "mother* perception*" OR "mother* expectation*" OR "mother* belief*" OR "mother* cognition*" OR "mother* schema*" OR “mother* criticism” OR “mother* self-efficacy” OR “mother* self-esteem” OR “mother* locus of control” OR “mother* empathy” OR “mother* role satisfaction” OR “mother* cognitive error*” OR "father* attitude*" OR "father* attribution*" OR "father* perception*" OR "father* expectation*" OR "father* belief*" OR "father* cognition*" OR "father* schema*" OR “father* criticism” OR “father* self-efficacy” OR “father* self-esteem” OR “father* locus of control” OR “father* empathy” OR “father* role satisfaction” OR “father* cognitive error*” OR "maternal attitude*" OR " maternal attribution*" OR " maternal perception*" OR " maternal expectation*" OR "maternal belief*" OR " maternal cognition*" OR " maternal schema*" OR “maternal criticism” OR “maternal self-efficacy” OR “maternal self-esteem” OR “maternal locus of control” OR “maternal empathy” OR “maternal role satisfaction” OR “maternal cognitive error*” OR "paternal attitude*" OR " paternal attribution*" OR "paternal perception*" OR " paternal expectation*" OR "paternal belief*" OR "paternal cognition*" OR "paternal schema*" OR “paternal criticism” OR “paternal self-efficacy” OR “paternal self-esteem” OR “paternal locus of control” OR “paternal empathy” OR “paternal role satisfaction” OR “paternal cognitive error*” OR “coparent*” OR “parent* alliance” |
