# Supplementary material for: Adult attention-deficit/hyperactivity symptoms and parental cognitions: a meta-analysis
Source: Front Psychiatry. 2024 Jan 9;14:1321078. doi: 10.3389/fpsyt.2023.1321078 (PMC10807045; doi:10.3389/fpsyt.2023.1321078)
Supplement: Supplementary file 2 [file Table_2.DOCX]

# Table S2

Quality assessment scores (1) and relevant findings of the studies

| First author, year | Sample characteristics | Relevant findings | Quality assessment | | | | | | | |
| --- | --- | --- | --- | --- | --- | --- | --- | --- | --- | --- |
|  |  |  | A/Q1 | A/Q2 | D/Q1 | D/Q2 | E/Q2 | E/Q2 | H/Q3 | Note |
| Banks, 2008 (2) | Community sample of mothers of preschool children (*N* = 80) | Women with high levels of ADHD symptoms reported lower parenting self-esteem, a more external parenting locus of control, and less effective disciplinary styles than women with lower levels of ADHD symptoms. | 1 | 2 | 1 | 3 | 1 | 1 | 1 | Strong everywhere except in D, where participants' knowledge of the study is not revealed, so the overall score in point D is moderate |
| Fabrikant-Abzug, 2023 (3) | Parents of school-aged children with ADHD (*N* = 199) | Parental ADHD symptoms were positively related to parental cognitive error | 1 | 5 | 1 | 1 | 1 | 1 | 1 | Strong everywhere except A2 |
| Johnston, 2018 (4) | Mothers and fathers of 5-13-year-old sons with and without ADHD (*N* = 156). | Parental inattention was unrelated to tolerance of misbehaviour, encouragement of child autonomy and parental empathy. Parental hyperactivity/impulsivity was positively related only to parental encouragement of child autonomy. | 1 | 5 | 1 | 1 | 1 | 1 | 1 | Strong everywhere but in A we do not know how many participants completed |
| Lindström, 2022 (5) | Parents of children with ADHD attending a psychoeducational program (*N* = 549) | At base-line, parents in the low and in the high ADHD strata did not differ in their causal attributions about the child’s behaviour. | 1 | 1 | 1 | 1 | 1 | 1 | 1 | Strong everywhere |
| Lowry, 2018 (6) | 79 mother-father dyads | Self-reported parental ADHD symptoms were negatively related to parental self-efficacy, positively related to caregiver strain, | 1 | 5 | 1 | 1 | 1 | 1 | 1 | Strong everywhere but in A we do not know how many participants completed |
| First author, year | Sample | Relevant findings | Quality assessment | | | | | | | |
|  |  |  | A/Q1 | A/Q2 | D/Q1 | D/Q2 | E/Q2 | E/Q2 | H/Q3 | Note |
| Moroney, 2017 (7) | Parents of 5–10 year-old children with and without ADHD (*N* = 205) | Parental ADHD was unrelated to parental criticism but showed a positive association with emotional over-involvement. | 1 | 1 | 1 | 1 | 1 | 1 | 1 | Strong everywhere |
| Ninowski, 2007 (8) | First-time expectant women (*N* = 86) | Maternal ADHD symptoms were related to less positive prenatal expectations regarding the infant and the future maternal role and lower maternal self-efficacy | 1 | 2 | 1 | 1 | 1 | 1 | 1 | Strong everywhere except in A where the second question is moderate |
| Park, 2019 (9) | Seventy-nine mothers of 6- to 11-year-old boys (N = 79) | Mothers’ ADHD symptoms were related to more child-responsibility attributions for negative behaviour and less child-responsibility attributions for positive behaviour. | 2 | 2 | 1 | 1 | 1 | 1 | 1 | Strong everywhere except in A moderate in both places |
| Psychogiou, 2007 (10) | Mothers of school-aged children (*N* = 100) | Parental ADHD symptoms were unrelated to parental criticism and emotional overinvolvement. | 1 | 2 | 1 | 1 | 1 | 1 | 1 | Strong everywhere except A/2 where it is moderate |
| Psychogiou, 2008 (11) | Mothers of school-aged children (*N* = 268) | Parental ADHD symptoms were negatively related to parental empathy. | 1 | 5 | 1 | 1 | 1 | 1 | 1 | Strong everywhere except A2, we do not know the proportion of selected persons and participants |
| Richards, 2014 (12) | Mothers of children with ADHD (*N* = 385) | Parental ADHD problems were unrelated to parental criticism. | 1 | 5 | 1 | 1 | 1 | 1 | 1 | strong everywhere except A/2, moderate |
| Sonuga-Barke, 2002 (13) | Mothers with 3-years-old children with ADHD (*N* = 83) | Mothers with high levels of ADHD reported lower levels of parental self-efficacy and satisfaction with the parental role than mothers with low levels of ADHD. | 1 | 1 | 1 | 1 | 1 | 1 | 1 | Strong everywhere |
|  |  |  |  |  |  |  |  |  |  |  |
| First author, year | Sample | Relevant findings | Quality assessment | | | | | | | |
|  |  |  | A/Q1 | A/Q2 | D/Q1 | D/Q2 | E/Q2 | E/Q2 | H/Q3 | Note |
| Watkins, 2009 (14) | Mothers of 6-month-old infants (N = 99) | Mothers with higher levels of ADHD symptoms described themselves as having less parental impact and lower parenting satisfaction. | 1 | 3 | 1 | 1 | 1 | 1 | 1 | Strong everywhere but weak at A 2, less than 60% agreed to the study |
| Williamson, 2016 (15) | Parents of 8- to 12-year-old boys with and without ADHD (*N =* 64 dyads) | Maternal but not paternal ADHD was negatively related to parenting alliance. | 1 | 5 | 1 | 1 | 1 | 1 | 1 | strong everywhere except A2 |
| Williamson, 2019 (16) | Mothers of 6-12 year-old children (*N* = 120) | Parental ADHD symptoms were negatively related to parental self-efficacy. | 3 | 1 | 1 | 1 | 1 | 1 | 1 | Strong everywhere except A1 |

# References

1. Evans N, Lasen M, Tsey K. Appendix A: effective public health practice project (EPHPP) quality assessment tool for quantitative studies. Syst Rev Rural Dev Res Charact Des Qual Engagem Sustain USA Springer. 2015;45–63.

2. Banks T, Ninowski JE, Mash EJ, Semple DL. Parenting behavior and cognitions in a community sample of mothers with and without symptoms of attention-deficit/hyperactivity disorder. J Child Fam Stud. 2008;17:28–43.

3. Fabrikant-Abzug G, Friedman L, Pfiffner L. Examining Relations Between Parent and Child Psychopathology in Children with ADHD: Do Parent Cognitions Matter? J Psychopathol Behav Assess. 2023 Mar;45(1):75–87.

4. Johnston C, Williamson D, Noyes A, Stewart K, Weiss MD. Parent and Child ADHD Symptoms in Relation to Parental Attitudes and Parenting: Testing the Similarity-Fit Hypothesis. J Clin CHILD Adolesc Psychol. 2018;47(1):S127–36.

5. Lindstrom T, Suttner A, Forster M, Bolte S, Hirvikoski T. Is Parents’ ADHD Symptomatology Associated With the Clinical Feasibility or Effectiveness of a Psychoeducational Program Targeting Their Children’s ADHD? J Atten Disord. 2022 Oct;26(12):1653–67.

6. Lowry LS, Schatz NK, Fabiano GA. Exploring parent beliefs and behavior: the contribution of ADHD symptomology within mothers and fathers. J Atten Disord. 2018;22(13):1255–65.

7. Moroney E, Tung I, Brammer WA, Peris TS, Lee SS. Externalizing outcomes of youth with and without ADHD: time-varying prediction by parental ADHD and mediated effects. J Abnorm Child Psychol. 2017;45:457–70.

8. Ninowski JE, Mash EJ, Benzies KM. Symptoms of attention-deficit/hyperactivity disorder in first-time expectant women: Relations with parenting cognitions and behaviors. Infant Ment Health J. 2007;28(1):54–75.

9. Park JL, Johnston C. Mothers’ Attributions for Positive and Negative Child Behavior: Associations With Mothers’ ADHD Symptoms. J Atten Disord. 2019;23(5, SI):475–86.

10. Psychogiou L, Daley DM, Thompson MJ, Sonuga-Barke EJ. Mothers’ expressed emotion toward their school-aged sons: Associations with child and maternal symptoms of psychopathology. Eur Child Adolesc Psychiatry. 2007;16:458–64.

11. Psychogiou L, Daley D, Thompson M, Sonuga-Barke E. Parenting empathy: Associations with dimensions of parent and child psychopathology. Br J Dev Psychol. 2008 Jun;26:221–32.

12. Richards J, Vasquez A, Rommelse N, Oosterlaan J, Hoekstra P, Franke B, et al. A Follow-Up Study of Maternal Expressed Emotion Toward Children With Attention-Deficit/Hyperactivity Disorder (ADHD): Relation With Severity and Persistence of ADHD and Comorbidity. J Am Acad CHILD Adolesc PSYCHIATRY. 2014 Mar;53(3):311–9.

13. Sonuga-Barke EJ, Daley D, Thompson M. Does maternal ADHD reduce the effectiveness of parent training for preschool children’s ADHD? J Am Acad Child Adolesc Psychiatry. 2002;41(6):696–702.

14. Watkins SJ, Mash EJ. Sub‐clinical levels of symptoms of attention‐deficit/hyperactivity disorder and self‐reported parental cognitions and behaviours in mothers of young infants. J Reprod Infant Psychol. 2009 Feb 1;27(1):70–88.

15. Williamson D, Johnston C. Marital and coparenting relationships: associations with parent and child symptoms of ADHD. J Atten Disord. 2016;20(8):684–94.

16. Williamson D, Johnston C. Maternal ADHD Symptoms and Parenting Stress: The Roles of Parenting Self-Efficacy Beliefs and Neuroticism. J Atten Disord. 2019 Mar;23(5):493–505.
